# Construct Validity of the Measurement Tools PH42 and I.ROC12 to Measure Positive Health in a General Population

**DOI:** 10.1007/s10728-025-00533-2

**Published:** 2025-07-31

**Authors:** Vera P. van Druten, Lenny M. W. Nahar-van Venrooij, Bea G. Tiemens, Dike van de Mheen, Esther de Vries, Margot J. Metz

**Affiliations:** 1 Jeroen Bosch Academy Research, Jeroen Bosch Hospital, ‘s-Hertogenbosch, The Netherlands; 2 Tranzo Scientific Centre for Care and Wellbeing, Tilburg University, Professor Cobbenhagenlaan 125, PO box 90153, 5000 LE Tilburg, The Netherlands; 3 Behavioural Science Institute, Radboud University, Nijmegen, The Netherlands; 4 Pro Persona Research, Renkum, The Netherlands; 5 GGz Breburg, Specialist Mental Health Care Organisation, Tilburg, The Netherlands

**Keywords:** Positive health, Recovery, Patient-centred outcomes research, Patient reported outcome measures, Measurement properties, Construct validity

## Abstract

**‘**Positive Health’ and ‘recovery’ seem to cover similar multidimensional health perspectives focussing on capabilities instead of incapabilities. The My Positive Health questionnaire and Individual Recovery Outcomes Counter were initially developed as dialogue tools, but nowadays also used as self-reported questionnaires. Structural validity of these dialogue tools was assessed in earlier research resulting in the 42-items Positive Health questionnaire (PH42) and 12-items Individual Recovery Outcomes Counter (I.ROC12). As a next step, we investigated their construct validity. An observational cross-sectional study was conducted in a representative general Dutch population (LISS-panel) determining (1) Coherence between the PH42 and I.ROC12 using correlation coefficients; (2) Convergent validity by testing hypotheses for PH42 and I.ROC12 with external health-related questions using correlation coefficients; (3) Discriminative validity for subgroups gender, age, educational level and healthcare use. (1) Nine out of twelve correlations between PH42 and I.ROC12 factors were substantial (> 0.5). (2) Hypotheses for PH42 and I.ROC12 factors with health-related questions were confirmed for 80% and 75%, respectively. (3) Scores on all factors increased (i.e., better health) from low to high educational level and decreased from no healthcare use to healthcare received from (medical) specialists. Only the factor physical health and functioning showed a continuous decrease in scores with increasing age. Women scored lower only on physical health and functioning. Convergent validity is adequate and discriminative validity is adequate for educational level and healthcare use supporting the conclusion that the PH42 and I.ROC12 are useful instruments to measure Positive Health in a general population.

**Supplementary Information:**

The online version contains supplementary material available at https://doi.org/10.1007/s10728-025-00533-2.

## Introduction

In 2011 Huber et al. published a new perspective on health as “the ability to adapt and self**-**manage in the face of social, physical, and emotional challenges” [1]**,** later called ‘Positive Health’ [2]. This perspective differs from the original WHO concept of health, because it focusses on capabilities instead of incapabilities, and people with disabilities or chronic diseases are no longer automatically seen as ‘not healthy’ [1, 3]. In mental healthcare, a comparable shift has taken place for the perspective called ‘recovery’, which next to clinical recovery includes social and personal recovery, respectively described as someone’s role(s) in society and as living a satisfying, hopeful, and meaningful life [4–6]. Although Positive Health and recovery were developed from different backgrounds, both perspectives focus on comparable multidimensional constructs [1, 4–6]. Positive Health was developed to promote a broad perspective on health in general while recovery was developed from a process-oriented perspective on recovery in mental healthcare. If these constructs cover similar aspects, a uniform construct to evaluate Positive Health across the entire healthcare system might be created.

For both perspectives, a dialogue tool was developed including a self-reported questionnaire to guide the conversation on health between patients and healthcare professionals and to stimulate self-reflection [2, 7]. An important question arising from daily practice is therefore whether the items from these dialogue tools can serve as self-reported questionnaires aiming to measure the Positive Health status in a general population. The dialogue tool My Positive Health questionnaire (MPH) consists of 42 items divided over six dimensions: bodily functions, mental wellbeing, meaningfulness, quality of life, social and societal participation and daily functioning [2]. The dialogue tool Individual Recovery Outcomes Counter (I.ROC) consists of 12 items divided over four dimensions: home, opportunity, people, and empowerment [7, 8] and is content-wise similar to the MPH dialogue tool.

Since the broader perspective of Positive Health became more important, the need for instruments to evaluate the effectiveness of working with this perspective increased [9, 10]. Therefore, the development and validation of appropriate measurement tools is essential to support and facilitate the evaluation of using the broader Positive Health perspective [9, 10]. This could be relevant given the high rates of somatic and psychological comorbidity [11]. However, no reference standard to measure Positive Health is available so far. Both the MPH and the I.ROC tools were initially developed as dialogue tools. Meanwhile, they were further investigated for measurement properties [7, 12]. For MPH, a shortened 17-items measurement questionnaire was developed and it was strongly related to happiness and self-rated health, and moderately related to autonomy, self-care, stability, personal growth and resilience [12, 13]. However, the response rate in this study was low and the preferred methodological approach for item reduction [14] was not applied. In contrast to the MPH dialogue tool, the I.ROC dialogue tool was already validated for measurement purposes in several English and Dutch mental healthcare practices showing good results in terms of validity and reliability for its use as a measurement tool. The I.ROC as a measurement tool was strongly related to the tools measuring symptoms of mental illness and social functioning, clinical outcomes of interventions and mental and societal functioning, moderately to strongly related to quality of life and personal recovery, and weakly to moderately related to a psychotic symptoms scale, and functional recovery [7, 15–18]. The I.ROC tool has been widely used in mental health settings for dialogue and measurement purposes [7, 15–19], but was not yet investigated in a general population.

Because of the conceptual similarities between the MPH and I.ROC as *dialogue* tools we hypothesised that the tools might be coherent. In our previous study [20], we concluded that the 42 items Of the MPH and the 12 items of the I.ROC can be used to measure Positive Health in a general population, but the distribution of the items over the dimensions was different from the dialogue tools. These measurement tools were called the PH42 and the I.ROC12. Both measurements tools (PH42 and I.ROC12) showed sufficient structural validity in general populations. Also, coherence exists between the total scores of the tools [20]. As a next step, using the COSMIN taxonomy as scientific guideline for developing and evaluating Patient-Reported Outcome Measures (PROMs) [21], it would be relevant to investigate their coherence and construct validity in greater depth.

The aim of this study was to assess the construct validity of the PH42 and the I.ROC12 by studying: (1) the coherence between both measurement tools in more detail; (2) the convergent validity of PH42 and I.ROC12 by comparing their scores with scores from general health questions; and (3) the discriminative validity of these tools by comparing scores between subgroups.

## Method

### Study Design

An observational cross-sectional research design was used to investigate construct validity of the PH42 and I.ROC12 measurement tools in a representative cohort from the general Dutch population.

### Participants

The LISS panel (Longitudinal Internet Studies for the Social sciences) is a panel for measurement and experimentation in the social sciences project (MESS) of CentERdata (http://www.lissdata.nl) [18, 22]. The LISS panel is representative for the Dutch population consisting of 7,500 individuals from 5000 households. The LISS panel is composed based on a true probability distribution sample of households drawn from the population register by Statistics Netherlands [22]. At least one person in the household should master the Dutch Language. If necessary for participation, households were provided with a computer and internet connection by CentERdata. The LISS panel has been in full operation since 2007 and all data are made available to researchers and policymakers (upon request) through the LISS data archive (www.lissdata.nl). For this study, a random selection of Dutch adults (≥ 18 years) from the LISS panel was included (n = 2457).

### Data Collection

LISS panel members complete online questionnaires every month and are paid for each completed questionnaire. In November 2020, they completed a LISS Core study module about health, and filled out the MPH and I.ROC questionnaires (https://www.lissdata.nl/research/liss-core-study). This dataset (including 2,457 respondents) was also used in earlier research of van Druten et al. [20]. For this study a minimum sample size of 50 respondents per subgroup was needed [14].

### Measurements

The 42-items of PH42 are statements on an 11-point scale where 0 means ‘totally disagree’ and 10 ‘totally agree’, higher scores represent better experienced Positive Health. The I.ROC12 consists of 12 items with a six-point scale from 1 ‘never’ to 6 ‘all the time’; higher scores represent better experienced health and wellbeing. In earlier research of van Druten et al. [20], research was conducted into the factor structure of the tools in the general population using the same LISS cohort for both tools. Exploratory factor analyses of the PH42 resulted in a six-factor structure: ´Acceptance, meaningfulness and satisfaction with life’, ‘Physical health and functioning’, ‘Self-management’, ‘Social network and societal roles’, ‘Personal development’ and ‘Cognition’. Internal consistencies of four factors were good (> 0.7) and for the factors, ‘Acceptance, meaningfulness and satisfaction with life’ and ‘Social network and societal roles’, high (> 0.90). In confirmatory factor analyses, the factor structure was confirmed with acceptable fit indices [20]. Exploratory factor analyses of the I.ROC12 resulted in a two-factor solution with acceptable fit indices and good internal consistency (0.7–0.90): ‘Wellbeing, control, network and meaningfulness’ and ‘Health, safety and abilities’ [20]. See the Online appendix for all factors and included items of PH42 and I.ROC12.

The LISS Core study aims to measure changes in people’s life course and effects of societal changes and policy. The core study consists of eight themes: ‘health’, ‘politics and values’, ‘religion and ethnicity’, ‘social integration and leisure’, ‘family and household’, ‘work and schooling’, ‘personality’, ‘economic situation assets, income, housing’. Four health-related questions of the LISS Core study were available to use as external questions for hypothesis testing in this study: (1) ‘How would you describe your health, generally speaking?’ (question from the Health-monitor of centERdata) which scores on a five-point scale from 1 ‘poor’ to 5 ‘excellent’. (2) The sum score of mental health questions: ‘I felt very anxious, ‘I felt so down that nothing could cheer me up’, ‘I felt calm and peaceful’, ‘I felt depressed and gloomy’ and ‘I felt happy’ (questions from the Mental Health Inventory 5 (MHI-5) [23]) which score on a six-point scale from 1 ‘never’ to 6 ‘continuously’. (3) ‘Did your physical health or emotional problems hinder your daily activities over the past month, for instance in going for a walk, walking upstairs, dressing yourself, washing yourself, visiting the toilet?’ (question from the Health-monitor of centERdata), and (4) ‘To what extent did your physical health or emotional problems hinder your work over the past month, for instance in your job, in housekeeping, in taking care of the children, in doing volunteer work, or in school?’ (question from the Health-monitor of centERdata). Questions 3 and 4 score on a five-point scale from 1 ‘not at all’ to 5 ‘very much’.

### Statistical Analysis

#### Characteristics of the Respondents

Characteristics of the respondents were presented depending on their distribution by mean, median or frequencies and their deviation measurements. Educational level was categorised into high, middle and low level corresponding to the categorisation used by Statistics Netherlands [24].

#### Coherence Between Both Tools

Coherence between both tools was assessed by calculating the Pearson and Spearman correlation coefficients between the factors of PH42 and I.ROC12 [20]. Correlation coefficients of > 0.3 were considered moderate and > 0.5 substantial correlations [25, 26].

#### Convergent Validity

Convergent validity was assessed by comparing factor scores separately with the external health-related questions from the LISS Core study. Based on the literature, nine hypotheses about the coherence between the factor scores and the external health-related questions were formulated beforehand by the research team. A wide range of potential associations was extensively discussed within the research team and converged in the nine hypotheses mentioned below. As outlined by the COSMIN guidelines the hypotheses were tested to assess construct validity, using Pearson and Spearman correlation coefficients. Correlation coefficients of > 0.3 were considered moderate and > 0.5 substantial correlations [25, 26]. For hypothesis testing 75% should be confirmed for adequate construct validity [14]. The nine hypotheses were:

1. The answer to the question’how would you describe your health generally speaking?’ is correlated > 0.5 to the factor Acceptation, meaningfulness and satisfaction with life (PH42).
2. The answer to the question’how would you describe your health generally speaking?’ is correlated > 0.5 to the factor Physical health and functioning (PH42).
3. The answer to the question’how would you describe your health generally speaking?’ is correlated > 0.5 to the factor Health, safety and abilities (I.ROC12).
4. The sum score of the answer to the following questions: ‘I felt very anxious’, ‘I felt so down that nothing could cheer me up’, ‘I felt calm and peaceful’, ‘I felt depressed and gloomy’.’ is correlated > 0.5 to the factor Acceptation, meaningfulness and satisfaction with life (PH42).
5. The sum score of the answer to the following questions: ‘I felt very anxious’, ‘I felt so down that nothing could cheer me up’, ‘I felt calm and peaceful’, ‘I felt depressed and gloomy’.’ is correlated > 0.5 to the factor Wellbeing, control, network and meaningfulness (I.ROC12).
6. The answer to the question ‘did your physical health or emotional problems hinder your daily activities over the past month, for instance in going for a walk, walking upstairs, dressing yourself, washing yourself, visiting the toilet?’ is correlated > 0.5 to the factor Physical health and functioning (PH42).
7. The answer to the question ‘did your physical health or emotional problems hinder your daily activities over the past month, for instance in going for a walk, walking upstairs, dressing yourself, washing yourself, visiting the toilet?’ is correlated > 0.5 to the factor Health, safety and abilities (I.ROC12).
8. The answer to the question ‘to what extent did your physical health or emotional problems hinder your work over the past month, for instance in your job, the housekeeping, taking care of the children, doing volunteer work. or in school?’ is correlated > 0.5 to the factor Physical health and functioning (PH42).
9. The answer to the question ‘to what extent did your physical health or emotional problems hinder your work over the past month, for instance in your job, the housekeeping, taking care of the children, doing volunteer work. or in school?’ is correlated > 0.5 to the factor Wellbeing, control, network and meaningfulness (I.ROC12).

#### Discriminative Validity

Discriminative validity was assessed by comparing subgroups classified by gender (men/women), age (categories of 10 years), educational level (categories low, middle, high), and healthcare use (categories none, general practitioner and medical specialist) on each factor score and the total scores of both tools (PH42 and I.ROC12). Visiting a medical specialist was rated as most intensive healthcare use. Descriptives (mean, median, standard deviation, minimum, maximum, percentiles 5, 25, 75 and 95) of the factor scores were calculated per subgroup. To assess differences for the numeric data between two subgroups the independent t-test was applied for normally distributed data and the Mann–Whitney U test for not normally distributed data; for > 2 subgroups One-way ANOVA was applied for normally distributed data and the Kruskal–Wallis test for not normally distributed data. A *p*-value < 0.05 was considered statistically significant. Analyses were conducted in IBM SPSS version 27 [27].

### Ethical Considerations

The study was conducted in accordance with current laws, regulations, and principles of the Declaration of Helsinki. This study was part of the LISS panel study, a non-commercial online longitudinal research panel, operational since 2007. Ethical approval was waived by the METC Brabant (Tilburg, the Netherlands, study number NW2024-15). Informed consent was given by all participants to be included as a member in the LISS-panel and again for each monthly questionnaire separately. Respondents were informed about the goal of the study prior to giving consent and completion of the questionnaire. Participation was voluntary. The General Data Protection Regulation (GDPR) was followed. All data from the LISS panel are anonymised and open access available upon request for researchers and policymakers.

## Results

### Characteristics of the Respondents

The response rate was high (76%) with 777 respondents not responding. Twelve respondents were excluded because they did not fill out the questionnaires completely, leaving 2,457 respondents for the analyses. Characteristics of the respondents are shown in Table 1. The subgroups ranged between 64 (subgroup 18–20 years) and 1,327 (subgroup gender: women) included respondents.

**Table 1:** Characteristics of the respondents

		Metrics	Total (n = 2,457)
			n (% of total) unless stated otherwise in the metrics column
Gender (W)			1,327 (54)
Age		Mean ± SD	53.3 ± 17.8
Educational level^a^	Low		603 (24.5)
	Middle		867 (35.3)
	High		980 (39.9)
	Missing		7 (0.3)
Personal monthly income	Euro	Median (IQR)	3,000 (2,205)
	Missing		133 (5.4)
Healthcare use^b^	None		751 (30.6)
	General practitioner		705 (28.7)
	Medical specialist		977 (39.8)
	Missing		24 (1.0)

*n* number, *W* women, *SD* standard deviation, *IQR* interquartile range

^a^Educational level. Low: primary education, prevocational secondary education, first three years of senior general secondary education, first three years of pre-university education and senior secondary vocational education level 1. Middle: four or five years of senior general secondary education, four, five or six years of pre-university education and senior secondary vocational education, level 2, 3 or 4. High: higher vocational education and university education

^b^Healthcare use. None: no visit to the general practitioner or medical specialist. General practitioner: one or more visits to the general practitioner. Medical specialist: one or more visits to a medical specialist at the hospital, or to a psychiatrist, psychologist, or psychotherapist

### Coherence Between Both Tools

Nine out of twelve correlations between the factors of PH42 and I.ROC12 were considered substantial (between 0.519 and 0.734). The other three correlations were considered moderate (between 0.434 and 0.463). For details see Table 2.

**Table 2:** Coherence between the factors of PH42 and I.ROC12 calculated with Pearson and Spearman correlation coefficients

			PH42^a^					
			Acceptance, meaningfulness and satisfaction with life	Physical health and functioning	Self-management	Social network and societal roles	Personal development	Cognition
I.ROC12^a^	Wellbeing, control, network and meaningfulness	Pearson/Spearman	0.767/0.734	0.542/0.519	0.578/0.551	0.704/0.662	0.566/0.555	0.467/0.434
	Health, safety and abilities	Pearson/Spearman	0.655/0.626	0.693/0.666	0.620/0.580	0.568/0.528	0.456/0.456	0.497/0.463

^a^PH42 and I.ROC12 factors from factor analyses [20]

All Spearman correlation coefficients were statistically significant (< 0.05)

### Convergent Validity

For the PH42, four out of five (80%) hypothesised substantial correlations with the external health-related questions were confirmed. The other correlation was moderate (0.479). For the I.ROC12, three out of four (75%) hypothesised substantial correlations were confirmed. The other one was moderate (-0.369) (see Table 3).

**Table 3:** Convergent validity. Hypothesised and calculated correlations of PH42, I.ROC12 with the external health-related questions of the LISS Core study

LISS Core study questions	PH42/I.ROC12 factors^a^		Hypothesised correlation	Calculated Pearson/Spearman correlation coefficient	Were hypotheses confirmed?
How would you describe your health generally speaking?	PH42	Acceptation, meaningfulness and satisfaction with life	> 0.5	0.488/0.479	No
	PH42	Physical health and functioning	> 0.5	0.681/0.673	Yes
	I.ROC12	Health, safety and abilities	> 0.5	0.532/0.515	Yes
Sum scores of the questions: ‘I felt very anxious’, ‘I felt so down that nothing could cheer me up’, ‘I felt calm and peaceful’, ‘I felt depressed and gloomy’	PH42	Acceptation, meaningfulness and satisfaction with life	> 0.5	0.734/0.680	Yes
	I.ROC12	Wellbeing, control, network and meaningfulness	> 0.5	0.623/0.574	Yes
To what extent did your physical health or emotional problems hinder your daily activities over the past month, for instance in going for a walk, walking upstairs, dressing yourself, washing yourself, visiting the toilet?	PH42	Physical health and functioning	> − 0.5	− 0.602/− 0.567	Yes
	I.ROC12	Health, safety and abilities	> − 0.5	− 0.538/− 0.500	Yes
To what extent did your physical health or emotional problems hinder your work over the past month, for instance in your job, the housekeeping, taking care of the children, doing volunteer work or in school?	PH42	Physical health and functioning	> − 0.5	− 0.568/− 0.528	Yes
	I.ROC12	Wellbeing, control, network and meaningfulness	> − 0.5	− 0.396/− 0.369	No

^a^PH42 and I.ROC12 factors from factor analyses [20]

All correlations were statistically significant (*P* < 0.001)

### Discriminative Validity

Factor sum scores for all factors and total scores were interpreted as normally distributed. No differences in factor scores were seen between men and women except for the PH42 factor ‘Physical health and functioning’; women scored significantly lower on this factor (see Tables 4 and 5 and Supplementary Table 1). Differences were seen between age on all factors of PH42 and I.ROC12, but only for the PH42 factor ‘Physical health and functioning’ a continuous decrease in scores was seen: mean and median scores decreased with increasing age (see Tables 4 and 5 and Supplementary Table 2). Furthermore, differences in scores were seen between educational levels for all factors of PH42 and I.ROC12: mean and median scores increased with higher educational level (see Tables 4 and 5 and Supplementary Table 3). Finally, for all factors of both tools the scores were different between different levels of healthcare use: mean scores decreased from no healthcare use to receiving healthcare from medical specialists (see Tables 4 and 5 and Supplementary Table 4). See Supplementary figures for differences between all subgroups of age, educational level and healthcare use.

**Table 4:** Total scores and sum scores per factor of PH42 by subgroups

		Acceptance, meaningfulness, and satisfaction with life (min–max score on the factor 0–130)	Physical health and functioning (min–max score on the factor 0–80)	Self-management (min–max score on the factor 0–70)	Social network and societal roles (min–max score on the factor 0–80)	Personal development (min–max score on the factor 0–30)	Cognition (min–max score on the factor 0–30)	Total score (min–max score on the total score 0–420)
		Mean ± SD	Mean ± SD	Mean ± SD	Mean ± SD	Mean ± SD	Mean ± SD	Mean ± SD
Gender^a^	Men (n = 1130)	99.0 ± 20.8	58.8 ± 12.7	58.0 ± 8.5	62.6 ± 11.5	21.8 ± 4.7	23.8 ± 4.3	323.9 ± 54.2
	Women (n = 1327)	97.8 ± 20.0	56.4 ± 13.1	58.3 ± 8.7	63.1 ± 11.3	21.9 ± 4.80	23.9 ± 4.3	321.4 ± 52.7
	*P*	0.161	< 0.001	0.413	0.225	0.694	0.501	0.244
Age^b^	18–20 (n = 64)	89*.*5 ± 24.6	58.4 ± 12.8	53.1 ± 10.1	57.8 ± 14	21.2 ± 5.3	23.4 ± 4.3	303.3 ± 62.9
	21–30 (n = 294)	98*.*8 ± 18.8	61.6 ± 10.5	57.3 ± 8.9	63.5 ± 10.9	23.6 ± 4	24.2 ± 3.9	329.0 ± 49.1
	31–40 (n = 320)	98*.*2 ± 21.5	58.4 ± 13.2	58.1 ± 9	62.5 ± 11.8	23.1 ± 4.4	24.1 ± 4.5	324.3 ± 56.2
	41–50 (n = 337)	95*.*8 ± 21.6	57.5 ± 13.3	57.5 ± 8.6	61.7 ± 11.3	21.6 ± 4.4	23.6 ± 4.5	317.7 ± 54.7
	51–60 (n = 442)	98*.*9 ± 20.2	57.5 ± 12.8	59 ± 8.2	62.9 ± 11.7	21.9 ± 4.6	24 ± 4	324.2 ± 52.2
	61–70 (n = 532)	101*.*2 ± 19.3	57.6 ± 12.7	59.6 ± 8.3	64 ± 11.3	21.8 ± 4.8	24.1 ± 4.1	328.3 ± 52.0
	71–80 (n = 381)	98*.*7 ± 19.0	55.0 ± 13.0	57.8 ± 8.3	63.1 ± 10.5	20.6 ± 4.9	23.4 ± 4.3	318.6 ± 51.6
	81 + (n = 87)	92.6 ± 22.8	49.7 ± 14.2	56.4 ± 8.9	62.2 ± 11.9	18.7 ± 6.2	22.1 ± 5.8	301.6 ± 57.1
	*P*	< 0.001	< 0.001	< 0.001	0.002	< 0.001	< 0.001	< 0.001
Educational level^b,c^	Low	95.3 ± 22.6	53.8 ± 14	56.4 ± 9.8	60.4 ± 12.9	20 ± 5.3	22.8 ± 4.9	54 ± 9.9
	Middle	98.2 ± 21.2	57.3 ± 12.7	58 ± 8.7	62.7 ± 11.7	21.8 ± 4.7	23.9 ± 4.3	55.5 ± 9
	High	100.4 ± 17.6	59.9 ± 11.5	59.4 ± 7.5	64.5 ± 9.8	23 ± 4.1	24.4 ± 3.7	56.2 ± 7.7
	*P*	< 0.001	< 0.001	< 0.001	< 0.001	< 0.001	< 0.001	< 0.001
Healthcare use^b,d^	None	102.7 ± 19.1	62.3 ± 10.9	59.5 ± 8.1	64.1 ± 11.0	22.5 ± 4.6	24.7 ± 3.9	335.7 ± 49.6
	General practitioner	100.3 ± 18.6	58.9 ± 11.9	58.9 ± 8.1	63.8 ± 10.7	22.2 ± 4.7	23.9 ± 4.1	328 ± 49.6
	Medical specialist	93.6 ± 21.6	52.7 ± 13.6	56.6 ± 9.2	61.3 ± 12.7	21.1 ± 4.9	23.1 ± 4.6	308.4 ± 55.8
	*P*	< 0.001	< 0.001	< 0.001	< 0.001	< 0.001	< 0.001	< 0.001

n: number, SD: standard deviation

^a^Independent T-test

^b^One-way Anova

^c^Educational level. Low: primary education, prevocational secondary education, first three years of senior general secondary education, first three years of pre-university education and senior secondary vocational education level 1. Middle: four or five years of senior general secondary education, four, five or six years of pre-university education and senior secondary vocational education, level 2, 3 or 4. High: higher vocational education and university education

^d^Healthcare use. None: no visit to the general practitioner or medical specialist. General practitioner: one or more visits to the general practitioner. Medical specialist: one or more visits to a medical specialist at the hospital, or to a psychiatrist, psychologist, or psychotherapist

**Table 5:** Total scores and sum scores per factor of I.ROC12 by subgroups

		Wellbeing*,* control*,* network and meaningfulness (min–max score on the factor 8–48)	Health*,* safety and abilities (min–max score on the factor 4–24)	Total score (min–max score on the total score 12–72)
		Mean (SD)	Mean (SD)	Mean (SD)
Gender^a^	Men (n = 1130)	35.3 ± 6.6	20.0 ± 2.9	55.4 ± 8.9
	Women (n = 1327)	35.6 ± 6.4	19.9 ± 3.0	55.5 ± 8.7
	*P*	0.307	0.285	0.692
Age^b^	18–20 (n = 64)	34.2 ± 7.3	19.4 ± 3.0	53.6 ± 9.9
	21–30 (n = 294)	35.7 ± 6.3	19.8 ± 2.9	55.5 ± 8.6
	31–40 (n = 320)	35.6 ± 6.7	19.6 ± 3.1	55.2 ± 9.2
	41–50 (n = 337)	35.4 ± 6.7	20.0 ± 3.0	55.4 ± 9.0
	51–60 (n = 442)	35.7 ± 6.6	20.1 ± 3.0	55.8 ± 8.9
	61–70 (n = 532)	35.9 ± 6.2	20.4 ± 2.8	56.4 ± 8.3
	71–80 (n = 381)	35.0 ± 6.4	20.0 ± 2.9	54.9 ± 8.7
	81 + (n = 87)	33.5 ± 6.3	18.7 ± 3.2	52.1 ± 8.7
	*P*	0.020	< 0.001	0.002
Educational level^b,c^	Low (n = 603)	34.4 ± 7.2	19.6 ± 3.3	54 ± 9.9
	Middle (n = 867)	35.5 ± 6.7	20 ± 3	55.5 ± 9
	High (n = 980)	36 ± 5.7	24.4 ± 3.7	56.2 ± 7.7
	*P*	< 0.001	< 0.001	< 0.001
Healthcare use^b,d^	None	36.7 ± 6.4	20.8 ± 2.6	57.5 ± 8.3
	General practitioner	36 ± 6.2	20.3 ± 2.7	56.4 ± 8.3
	Medical specialist	34.1 ± 6.5	19.1 ± 3.2	53.2 ± 9
	*P*	< 0.001	< 0.001	< 0.001

n: number, SD: standard deviation

^a^Independent T-test

^b^One-way Anova

^c^Educational level. Low: primary education, prevocational secondary education, first three years of senior general secondary education, first three years of pre-university education and senior secondary vocational education level 1. Middle: four or five years of senior general secondary education, four, five or six years of pre-university education and senior secondary vocational education, level 2, 3 or 4. High: higher vocational education and university education

^d^Healthcare use. None: no visit to the general practitioner or medical specialist. General practitioner: one or more visits to the general practitioner. Medical specialist: one or more visits to a medical specialist at the hospital, or to a psychiatrist, psychologist, or psychotherapist

## Discussion

The aim of our research was to assess construct validity of both the PH42 and the IROC12 by exploring coherence between the PH42 and I.ROC12 tools, and convergent and discriminative validity in order to assess whether the PH42 and I.ROC12 can be used as self-reported *measurement* instruments for Positive Health in a general population. Most of beforehand formulated hypotheses were confirmed when comparing the tools to external criteria (i.e., the health-related questions of the LISS Core study) implying sufficient to good convergent validity. Given the descending scores found for lower educational levels, more specialised healthcare use, and partly for gender and higher age, discriminative validity is considered adequate.

### Coherence Between the Tools

Results showed high coherence between the factors of PH42 and I.ROC12 which indicates that both tools measure similar constructs covering Positive Health aspects. In line with our hypotheses, the scores of both tools can be interpreted as a reflection of a similar construct with comparable dimensions. However, differences between the composition of the factors of the tools exist. The PH42 consists of six factors which are more sharply defined compared to the two broad factors of the I.ROC12. For example, specific health aspects that are covered in four factors of the PH42 (‘Acceptance, meaningfulness, and satisfaction with life’, ‘Self-management’, ‘Personal development’ and ‘Social network and societal roles’) are included in one less specific I.ROC12 factor (‘Wellbeing, control network, and meaningfulness’). Thus, aiming to measure specific dimensions such as ‘Physical health and functioning’, ‘Self-management’, ‘Personal development’, and ‘Cognition’ the PH42 gives more information than the broad factors of the I.ROC12. For measuring a total Positive Health score both PH42 and I.ROC12 are useful. Therefore, in practice both the shorter I.ROC12 containing two factors, or the PH42, which involves six specific factors, can be chosen depending on the specificity of the preferred outcomes and the feasibility and desirability of administering a longer or shorter questionnaire.

### Convergent Validity

As hypothesised, results showed strong associations (> 0.5) for the factors ‘Acceptance, meaningfulness and satisfaction with life’ and ‘Physical health and functioning’ of the PH42 and both I.ROC12 factors with the external general health questions of the LISS Core study. Most hypotheses were confirmed indicating that expected relationships for these factors with the external general health questions indeed exist. Noteworthy, of the factors correlated with the general health question of the LISS Core study, this question was stronger related to the factors ‘Physical health and functioning’ of the PH42 and ‘Health, safety and abilities’ of the I.ROC12 than to the factor ‘Acceptance, meaningfulness and satisfaction with life’ of the PH42 which is less focussed on physical health. It seems that people from the general Dutch population interpret health mainly as the physical part of health. Probably, in specific patient populations the correlation between the factors of PH42 and I.ROC12 with the general health questions could be different compared to the general population. This was also concluded in earlier research, which showed that interpretations of health differ between populations and no consensus could be made on one overall health concept [28].

### Discriminative Validity

Results showed that high educated people scored higher on all the PH42 and I.ROC12 factors compared to middle or low educated people. This is in line with earlier research among US citizens [29] and in a general Dutch and UK population [30]. Also, people from higher socio-economic status (SES) groups (who are generally higher educated) scored higher on general health scores than lower SES groups [31, 32].

Results showed that people consulting a medical specialist scored lower on all PH42 and I.ROC12 factors compared to people who did not use any healthcare. In earlier research, it was also found that people with chronic diseases reported lower health-related quality of life [30, 33]. However, it should be noted that in our study, the subgroup ‘visiting a medical specialist during the last year’ was rated as most intensive healthcare use, which does not automatically mean they all have a chronic health condition.

Hardly any differences in scores on the PH42 and I.ROC12 factors were found between men and women except for the PH42 factor ‘Physical health and functioning’, which is in contrast to earlier research where women in European countries also scored lower compared to men on self-care, cognition, relationships, vision, affect [34] and on quality of life [29, 35, 36]. However, the dimensions measured by our instruments differ from the dimensions described in the referred literature.

Differences in scores between age groups were seen on all PH42 and I.ROC12 factors, but in this study a continuous decrease from young to old age groups was only found for the PH42 factor ‘Physical health and functioning’ which aligns logically with aging. In contrast, other research showed decreased quality of life scores from young to old age groups [29, 36], lower scores on cognition and increased scores on ongoing thoughts about future-self [37], and on meaningfulness [38] among older people. However, within this last mentioned study [38] no differences were found for satisfaction with life which is in line with our findings. Different instruments and targeted constructs might be an explanation for these discrepant results.

### Reflection on other Measurement Tools

Recently, more measurement tools are being and have been developed to measure Positive Health or other broad perspectives on health, next to the PH42 and I.ROC12. For example, the 17-items Positive Health questionnaire (PH17) and the Context-sensitive Positive Health Questionnaire (CPHQ) [12, 39]. The CPHQ used Positive Health as a basis but aligned the questionnaire with another theoretical framework, the capability approach; items about context specific aspects (for example feeling (dis)advantaged because of religion) were added [39]. Both the PH42 and the PH17 were developed among a general Dutch population, however, for the PH17 development study [12] the response rate was low and the population was not representative. A recent (unpublished) study focussed on item reduction, based on another methodological approach than was used to shorten the PH17, resulting in a 22-items PH questionnaire [40]. It is of interest for future research to compare the various measurement tools for their measurement properties, including their specific aims, context and construct. Most likely, some of the instruments complement each other, while others show overlap.

### Limitations and Strengths

The strengths of this research is the large study population (n = 2,457), high response rate (76%) and the efforts to include a representative group of the general Dutch population. Nevertheless, there may be some degree of selection bias in this study. First, because migrants may be underrepresented as one member of the household had to master the Dutch language. Second, members of the LISS-panel had to be able to use a computer [22]. However, if selection bias is present, the expectation is that it was limited and had small effects on the results.

## Conclusion

The results indicate that Positive Health and recovery cover similar aspects as the scores of the measurement tools show high coherence. Convergent validity was adequate for both tools. Discriminative validity was also adequate for educational level and healthcare use subgroups; for age and gender this remains unclear. The results support the conclusion from our earlier research that both the PH42 and I.ROC12 tools are suited to measure Positive Health in a general population. Depending on the purposes, a short and comprehensible tool (I.ROC12) or a longer and more detailed measurement tool (PH42) may be preferred. In future research, we recommend comparing the various available measurement tools in the field of Positive Health for their measurement properties, including their specific aims, context and construct.

## Supplementary Information

Below is the link to the electronic supplementary material.

Supplementary file1 (DOCX 1023 KB)

Supplementary file2 (PDF 309 KB)

Supplementary file3 (DOCX 29 KB)

## Data Availability

All data from the LISS panel are anonymised and available upon request for researchers and policymakers. For more information see: https://www.lissdata.nl/access-data.
