# Supplementary material for: Construct Validity of the Measurement Tools PH42 and I.ROC12 to Measure Positive Health in a General Population
Source: Health Care Anal. 2025 Jul 31;34(3):624–42. doi: 10.1007/s10728-025-00533-2 (PMC13675334; doi:10.1007/s10728-025-00533-2)
Supplement: Supplementary file 1 [file 10728_2025_533_MOESM2_ESM.pdf]

Supplementary table 1: Total scores and sum scores per factor of PH42 by gender (men/women)

Total scores and sum scores per factor of PH42 by gender (men/women)

	Acceptance, meaningfulness, and satisfaction with life[1, 2] (min-max score on the factor 0-130)		Physical health and functioning (min-max score on the factor 0-80)[1, 2]			Self-management[1, 2] (min-max score on the factor 0-70)		Social network and societal roles[1, 2] (min-max score on the factor 0-80)		Personal development[1, 2] (min-max score on the factor 0-30)		Cognition[1, 2] (min-max score on the factor 0-30)		Total score[1, 2] (min-max score on the total score 0-420)	
	Men n=1130	Women n=1327	Men n=1130	Women n=1327	*P*[3]	Men n=1130	Women n=1327	Men n=1130	Women n=1327	Men n=1130	Women n=1327	Men n=1130	Women n=1327	Men n=1130	Women n=1327
Mean	99.0	97.8	58.8	56.4	<0.001	58.0	58.3	62.6	63.1	21.8	21.9	23.8	23.9	323.9	321.4
Median	103	101	61	58		59	60	64	64	2	23	24.	24	331	328
SD	20.8	20.0	12.7	13.1		8.5	8.7	11.5	11.3	4.7	4.8	4.3	4.3	54.2	52.7
Min	9	19	7	7		22	18	4	10	3	4	6	2	119	104
Max	130	130	80	80		70	70	80	80	30	30	30	30	420	420
Percentile 5	59	60	36	33		41	41	41	42	13	13	15	1	215	225
Percentile 25	89	87	52	48		53	54	56	57	19	19	21	22	29	293
Percentile 75	113	112	67	66		65	65	71	71	25	25	27	27	362	358
Percentile 95	128	127	78	75		70	70	79	79	29	29	30	30	401	396

Total scores and sum scores per factor of I.ROC12 by gender (men/women)

	Wellbeing, control, network and meaningfulness[1, 2] (min-max score on the factor 8-48)		Health, safety and abilities[1, 2] (min-max score on the factor 4-24)		Total score[1, 2] (min-max score on the total score 12-72)	
	Men n=1130	Women n=1327	Men n=1130	Women n=1327	Men n=1130	Women n=1327
Mean	35.3	35.6	20.0	19.9	55.4	55.5
Median	36	36	20	20	56	56
SD	6.6	6.4	2.9	3.0	8.9	8.7
Min	10	13	8	7	21	2
Max	48	48	24	24	72	72
Percentile 5	2	23	14	14	39	39
Percentile 25	31	31	18	18	50	50
Percentile 75	40	40	22	22	61	62
Percentile 95	46	46	24	24	69	68

1 PH42 and I.ROC factors from factor analyses [1].

2 No significant differences (<0.05) were seen.

3 Independent T-test.

Men (n=1,130), women (n=1,327).

Supplementary table 2: Total scores and sum scores per factor of PH42 by age

Total scores and sum scores per factor of PH42 by age																		
	Acceptance, meaningfulness, and satisfaction with life (min-max score on the factor 0-130)									Physical health and functioning (min-max score on the factor 0-80)[1]								
	18 - 20 (n=64)	21-30 (n=294)	31-40 (n=320)	41-50 (n=337)	51-60 (n=442)	61-70 (n=532)	71-80 (n=381)	81+ (n=87)	*P*[2]	18 - 20 (n=64)	21-30 (n=294)	31-40 (n=320)	41-50 (n=337)	51-60 (n=442)	61-70 (n=532)	71-80 (n=381)	81+ (n=87)	*P*[2]
Mean	89.5	98.8	98.2	95.8	98.9	101.2	98.7	92.6	<0.001	58.4	61.6	58.4	57.5	57.5	57.6	55.0	49.7	<0.001
Median	92	102	102	100	103	104	102	95		60	63	61	59	60	60	56	50	
SD	24.6	18.8	21.5	21.6	20.2	19.3	19.0	22.8		12.8	10.5	13.2	13.3	12.8	12.7	13.0	14.2	
Min	29	30	9	12	15	40	37	19		18	22	17	7	7	14	14	18	
Max	127	130	130	130	130	130	130	130		80	80	80	80	80	80	80	80	
Percentile 5	42	64	60	53	60	62	65	53		38	39	34	35	33	33	32	22	
Percentile 25	71	89	89	87	89	91	88	75		48	56	50	50	50	50	4	40	
Percentile 75	109	112	112	109	113	115	112	111		67	69	68	67	66	66	60	59	
Percentile 95	125	124	128	127	127	129	128	129		79	77	78	76	76	76	75	74	
	Self-management (min-max score on the factor 0-70)[1]									Social network and societal roles (min-max score on the factor 0-80)[1]								
Mean	53.1	57.3	58.1	57.5	59	59.6	57.8	56.4	<0.001	57.8	63.5	62.5	61.7	62.9	64	63.1	62.2	0.002
Median	53.5	58	60	59	60	61	59	57		60.5	65	64	64	64	65	64	64	
SD	10.1	8.9	9	8.6	8.2	8.3	8.3	8.9		14	10.9	11.8	11.3	11.7	11.3	10.5	11.9	
Min	20	23	22	18	23	22	23	31		18	7	4	10	15	24	24	27	
Max	69	70	70	70	70	70	70	70		78	80	80	80	80	80	80	80	
Percentile 5	36	40	40	40	42	45	44	37		29	44	40	40	42	42	42	40	
Percentile 25	47	52	53	53	55	55	53	52		50	57	56	56	57	58	57	56	
Percentile 75	61	64	65	63	65	66	64	64		70	71	71	69	71	72	71	72	
Percentile 95	67	70	70	69	70	70	70	69		77	80	79	77	79	80	80	79	
	Personal development (min-max score on the factor 0-30)[1]									Cognition (min-max score on the factor 0-30)[1]								
Mean	21.2	23.6	23.1	21.6	21.9	21.8	20.6	18.7	<0.001	23.4	24.2	24.1	23.6	24	24.1	23.4	22.1	<0.001
Median	22	24	24	22	23	23	21	19		24	25	25	24	24	24	24	23	
SD	5.3	4	4.4	4.4	4.6	4.8	4.9	6.2		4.3	3.9	4.5	4.5	4	4.1	4.3	5.8	
Min	8	9	5	5	4	3	6	5		11	10	9	5	2	6	9	6	
Max	30	30	30	30	30	30	30	30		30	30	30	30	30	30	30	30	
Percentile 5	10	15	15	14	14	13	12	7		14	17	15	15	17	16	15	11	
Percentile 25	19	22	21	19	19	18	18	15		21	22	22	21	22	22	21	18	
Percentile 75	25	27	26	25	25	25	24	23		26	27	27	27	27	27	27	27	
Percentile 95	28	30	30	28	29	29	28	28		30	30	30	30	30	30	30	30	
	Total score (min-max score on the total score 0-420)[1]																	
Mean	303.3	329.0	324.3	317.7	324.2	328.3	318.6	301.6	<0.001									

Median	314.5	331	332	327	33	334	325	306										
SD	62.9	49.1	56.2	54.7	52.2	52.0	51.6	57.1										
Min	136	123	119	104	124	140	167	119										
Max	407	420	420	420	420	420	420	420										
Percentile 5	190	235	217	214	221	224	226	203										
Percentile 25	273	307	299	291	295	299	289	266										
Percentile 75	343	367	362	356	361	365	356	339										
Percentile 95	395	395	399	401	399	400	395	388										
Total scores and sum scores per factor of I.ROC12 by age																		
	Wellbeing, control, network, and meaningfulness (min-max score on the factor 8-48)[1]									Health, safety, and abilities (min-max score on the factor 4-24)[1]								
	18 - 20 (n=64)	21-30 (n=294)	31-40 (n=320)	41-50 (n=337)	51-60 (n=442)	61-70 (n=532)	71-80 (n=381)	81+ (n=87)	*P*[2]	18 - 20 (n=64)	21-30 (n=294)	31-40 (n=320)	41-50 (n=337)	51-60 (n=442)	61-70 (n=532)	71-80 (n=381)	81+ (n=87)	
Mean	34.2	35.7	35.6	35.4	35.7	35.9	35.0	33.5	0.020	19.4	19.8	19.6	20.0	20.1	20.4	20.0	18.7	<0.001
Median	33.5	36.5	36	36	36	36	35	34		20	20	20	20	21	21	20	19	
SD	7.3	6.3	6.7	6.7	6.6	6.2	6.4	6.3		3.0	2.9	3.1	3.0	3.0	2.8	2.9	3.2	
Min	17	14	14	13	10	13	18	13		12	9	9	7	8	8	9	10	
Max	48	48	48	48	48	48	48	46		24	24	24	24	24	24	24	24	
Percentile 5	21	24	23	23	22	24	24	22		13	14	14	14	14	15	15	11	
Percentile 25	29	32	31	31	32	32	30	30		17	18	18	18	19	19	18	16	
Percentile 75	40	40	40	40	40	40	40	37		22	22	22	22	22	22	22	21	
Percentile 95	46	45	48	46	46	46	45	45		24	24	24	24	24	24	24	23	
	Total score (min-max score on the total score 12-72)[1]																	
Mean	53.6	55.5	55.2	55.4	55.8	56.4	54.9	52.1	0.002									
Median	54	56.5	56.5	57	57	57	56	53										
SD	9.9	8.6	9.2	9.0	8.9	8.3	8.7	8.7										
Min	30	24	25	25	24	21	30	23										
Max	71	72	72	72	72	72	72	69										
Percentile 5	36	40	37	39	38	42	39	36										
Percentile 25	47	50	50	50	51	51	49	47										
Percentile 75	61	61	61	62	62	62	61	58										
Percentile 95	69	68	71	68	69	69	68	66										
Mean	53.6	55.5	55.2	55.4	55.8	56.4	54.9	52.1										

1 PH42 and I.ROC factors from factor analyses [1].

2 One-way Anova.

Supplementary table 3: Total score and sum scores per factor of PH42 by educational level

Total scores and sum scores per factor of PH42 by educational level

	Acceptance, meaningfulness, and satisfaction with life (min-max score on the factor 0-130)[1]				Physical health and functioning (min-max score on the factor 0-80)[1]				Self-management (min-max score on the factor 0-70)[1]				Social network and societal roles (min-max score on the factor 0-80)[1]			
	Low (n=603)	Middle (n=867)	High (n=980)	*P*[2]	Low (n=603)	Middle (n=867)	High (n=980)	*P*[2]	Low (n=603)	Middle (n=867)	High (n=980)	*P*[2]	Low (n=603)	Middle (n=867)	High (n=980)	*P*[2]
Mean	95.3	98.2	100.4	<0.001	53.8	57.3	59.9	<0.001	56.4	58	59.4	<0.001	60.4	62.7	64.5	<0.001
Median	100	102	103		55	59	62		58	59	60		63	64	65	
SD	22.6	21.2	17.6		14.5	12.7	11.5		9.8	8.7	7.5		12.9	11.7	9.8	
Min	24	12	9		9	7	7		18	20	22		19	10	4	
Max	130	130	130		80	80	80		70	70	70		80	80	80	
Percentile 5	53	57	68		28	35	38		38	41	46		35	41	47	
Percentile 25	82	88	91		44	50	54		51	53	55		53	56	60	
Percentile 75	113	113	113		64	66	67		64	65	65		71	71	72	
Percentile 95	128	128	125		76	76	76		70	70	70		79	80	79	

	Personal development (min-max score on the factor 0-30)[1]				Cognition (min-max score on the factor 0-30)[1]				Total score (min-max score on the total score 0-420)[1]			
Mean	20	21.8	23	<0.001	22.8	23.9	24.4	<0.001	308.7	321.9	331.7	<0.001
Median	20	22	24		23	24	25		315	329	336	
SD	5.3	4.7	4.1		4.9	4.3	3.7		59.9	54.3	45.8	
Min	3	5	5		2	5	9		104	119	119	
Max	30	30	30		30	30	30		420	420	420	
Percentile 5	10	13	15		14	15	18		196	219	247	
Percentile 25	17	19	21		20	22	22		272	293	306	
Percentile 75	24	25	26		26	27	27		351	359	363	
Percentile 95	28	29	29		30	30	30		400	400	395	

Total scores and sum scores per factor of I.ROC12 by educational level

	Wellbeing, control, network, and meaningfulness (min-max score on the factor 8-48)[1]				Health, safety, and abilities (min-max score on the factor 4-24)[1]				Total score (min-max score on the total score 12-72)[1]			
	Low (n=603)	Middle (n=867)	High (n=980)	*P*[2]	Low (n=603)	Middle (n=867)	High (n=980)	*P*[2]	Low (n=603)	Middle (n=867)	High (n=980)	*P*[2]
Mean	34.4	35.5	36	<0.001	19.6	20	24.4	<0.001	54	55.5	56.2	<0.001
Median	35	36	37		20	20	25		55	56	57	
SD	7.2	6.7	5.7		3.3	3	3.7		9.9	9	7.7	
Min	13	10	14		7	9	9		21	24	25	
Max	48	48	48		24	24	30		72	72	72	
Percentile 5	22	23	26		13	14	18		37	39	42	
Percentile 25	29	31	32		18	18	22		47	50	51	

Percentile 75	40	40	40	22	22	27	61	62	62
Percentile 95	46	46	45	24	24	30	69	70	68

1 PH42 and I.ROC factors from factor analyses [1].

2 One-way Anova.

Low (n=603), Middle (n=867), High (n=980) educational level. Low: primary education, prevocational secondary education (‘VMBO’), first three years of senior general secondary education (‘HAVO’), first three years of pre-university education (‘VWO’) and senior secondary vocational education (‘MBO’) level 1. Middle: four or five years of senior general secondary education (‘HAVO’), four, five or six years of pre-university education (‘VWO’) and senior secondary vocational education (MBO) level 2, 3 or 4. High: higher vocational education (‘HBO’) and university education (‘WO’).

Supplementary table 4: Total score and sum scores per factor of PH42 by healthcare use

Total scores and sum scores per factor of PH42 by healthcare use

	Acceptance, meaningfulness, and satisfaction with life (min-max score on the factor 0-130)[1]				Physical health and functioning (min-max score on the factor 0-80)[1]				Self-management (min-max score on the factor 0-70)[1]				Social network and societal roles (min-max score on the factor 0-80)[1]			
	None (n=751)	GP (n=705)	MS (n=977)	*P*[2]	None (n=751)	GP (n=705)	MS (n=977)	*P*[2]	None (n=751)	GP (n=705)	MS (n=977)	*P*[2]	None (n=751)	GP (n=705)	MS (n=977)	*P*[2]
Mean	102.7	100.3	93.6	<0.001	62.3	58.9	52.7	<0.001	59.5	58.9	56.6	<0.001	64.1	63.8	61.3	<0.001
Median	106	103	97		64	61	54		61	60	58		65	64	64	
SD	19.1	18.6	21.6		10.9	11.9	13.6		8.1	8.1	9.2		11.0	10.7	12.1	
Min	12	15	9		15	18	7		20	22	18		18	19	4	
Max	130	130	130		80	80	80		70	70	70		80	80	80	
Percentile 5	65	65	51		42	37	27		44	42.3	40		44	42	40	
Percentile 25	93	90	82		56	52	44		55	55	51		58	58	54	
Percentile 75	117	113	109		70	67	63		65	65	63		72	72	70	
Percentile 95	129	128	125		78	76	73		70	70	70		80	79	78	

	Personal development (min-max score on the factor 0-30)[1]				Cognition (min-max score on the factor 0-30)[1]				Total score (min-max score on the total score 0-420)[1]			
Mean	22.5	22.2	21.1	<0.001	24.7	23.9	23.1	<0.001	335.7	328	308.4	<0.001
Median	23	23	22		25	24	24		342	334	316	
SD	4.6	4.7	4.9		3.9	4.1	4.6		49.6	49.6	55.8	
Min	5	3	5		9	2	5		136	140	104	
Max	30	30	30		30	30	30		420	420	420	
Percentile 5	14	13	13		17	16	15		238	231	204	
Percentile 25	20	20	18		22	22	21		309	301	278	
Percentile 75	26	25	25		27	27	26		370	360	346	
Percentile 95	29	29	29		30	30	30		404	400	389	

Total scores and sum scores per factor of I.ROC12 by health care use

	Wellbeing, control, network, and meaningfulness (min-max score on the factor 8-48)[1]				Health, safety, and abilities (min-max score on the factor 4-24)[1]				Total score (min-max score on the total score 12-72)[1]			
	None (n=751)	GP (n=705)	MS (n=977)	*P*[2]	None (n=751)	GP (n=705)	MS (n=977)	*P*[2]	None (n=751)	GP (n=705)	MS (n=977)	*P*[2]
Mean	36.7	36	34.1	<0.001	20.8	20.3	19.1	<0.001	57.5	56.4	53.2	<0.001
Median	37	37	35		21	21	20		59	57	54	
SD	6.4	6.2	6.5		2.6	2.7	3.2		8.3	8.3	9	
Min	10	12	13		8	10	7		21	23	24	
Max	48	48	48		24	24	24		72	72	72	
Percentile 5	25	25	22		16	15	13		42	42	37	

Percentile 25	33	32	30	19	19	17	53	51	48
Percentile 75	41	40	39	23	22	22	63	62	60
Percentile 95	47	46	44	24	24	24	70	69	67

1 PH42 and I.ROC factors from factor analyses [1].

2 One-way Anova.

None: no healthcare use in the past month. GP: visiting the general practitioner in the past month. MS: visiting a medical specialist in the past month.

[1] van Druten, V. P., Metz, M. J., Mathijssen, J. J. P., van de Mheen, D., van Vliet, M., Rudd, B., de Vries, E., & Nahar van - Venrooij, L. M. W. (2024). Measuring Positive Health using the My Positive Health (MPH) and Individual Recovery Outcomes Counter (I.ROC) Dialogue Tools: A Panel Study on Measurement Properties in a Representative General Dutch Population. *Applied research in quality of life*, *19*(5), 2825–2846. https://doi.org/10.1007/s11482-024-10356-3
