# Supplementary material for: Construct Validity of the Measurement Tools PH42 and I.ROC12 to Measure Positive Health in a General Population
Source: Health Care Anal. 2025 Jul 31;34(3):624–42. doi: 10.1007/s10728-025-00533-2 (PMC13675334; doi:10.1007/s10728-025-00533-2)
Supplement: Supplementary file 2 [file 10728_2025_533_MOESM1_ESM.docx]

**SUPPLEMENTARY FIGURES**

**Figures of distribution of the PH42 factor sum scores and total scores in age subgroups**

**Figures of distribution of the I.ROC12 factor sum scores and total scores in age subgroups**

**Figures of distribution of the PH42 factor sum scores and total scores in educational level subgroups**

**Figures of distribution of the I.ROC12 factor sum scores and total scores in educational level subgroups**

**Figures of distribution of the PH42 factor sum scores and total scores in healthcare use subgroups**

**Figures of distribution of the PH42 factor sum scores and total scores in healthcare use subgroups**
