# Supplementary material for: Construct Validity of the Measurement Tools PH42 and I.ROC12 to Measure Positive Health in a General Population
Source: Health Care Anal. 2025 Jul 31;34(3):624–42. doi: 10.1007/s10728-025-00533-2 (PMC13675334; doi:10.1007/s10728-025-00533-2)
Supplement: Supplementary file 3 [file 10728_2025_533_MOESM3_ESM.docx]

# APPENDIX

| **Factors and related items of the PH42 (van Druten et al., 2024)** | |
| --- | --- |
| Item number of the dialogue tool | Item |
| **Factor: Acceptance, meaningfulness and satisfaction with life** | |
| MW11 | I feel cheerful |
| MW12 | I accept myself for who I am |
| MW14 | I feel in control of my life |
| MF15 | I have a meaningful life |
| MF16 | In the morning, I look forward to the day ahead |
| MF18 | I feel confident about my own future |
| MF19 | I accept life as it comes |
| MF20 | I am grateful for what life offers me |
| QL22 | I enjoy my life |
| QL23 | I am happy |
| QL24 | I feel good |
| QL25 | I feel my life is well-balanced |
| QL26 | I feel safe |
|  | |
| **Factor: Physical health and functioning** | |
| BF1 | I feel healthy |
| BF2 | I feel fit |
| BF3 | I have no physical complaints or pain |
| BF4 | I sleep well |
| BF5 | I eat well |
| BF6 | I recover quickly after exercise, such as sports |
| BF7 | I find it easy to move, such as going up and down stairs, walking or cycling |
| DF41 | I am able to work in a job or do voluntary work |
|  | |
| **Factor: Self-management** | |
| QL28 | I have enough money to pay my bills |
| DF36 | I am well capable of looking after myself, for example, with regard to personal hygiene, getting dressed, shopping, cooking |
| DF37 | I know my limitations |
| DF38 | I know how I can look after my own health |
| DF39 | I am well capable of planning my day |
| DF40 | I am well capable of managing the money that I have each month |
| DF42 | I know how to apply for benefits or get assistance from official agencies when necessary |
|  | |
| **Factor: Social network and societal roles** | |
| QL27 | I am content about where and whom I live |
| SP29 | I am in good contact with other people |
| SP30 | Other people take me seriously |
| SP31 | I have people with whom I can do fun things |
| SP32 | I have people who support me when I need it |
| SP33 | I feel that I 'belong' in my environment |
| SP34 | I consider my job or other activities to be meaningful |
| SP35 | I am interested in what happens in society |
|  |  |
|  | |
| **Factor: Personal development** | |
| MW13 | I look for solutions to change difficult situations |
| MF17 | I have ideals that I would like to achieve |
| MF21 | I want to continue learning throughout my life |
|  | |
| **Factor: Cognition** | |
| MW8 | I am good at remembering things |
| MW9 | I am able to concentrate |
| MW10 | I am able to see, hear, talk, read |
| ^BF: bodily functions, MW: mental wellbeing, MF: meaningfulness, QL: quality of life, SP: social and societal participation, DF: daily functioning (Huber et al., 2016).^ | |

| **Factors and related items of the I.ROC12 (van Druten et al., 2024)** | |
| --- | --- |
| Item number of the dialogue tool | Item |
| **Factor: Health*,* safety and abilities** | |
| HO2 | In the past 3 months… How often have you felt you have the skills you need to look after yourself? |
| HO3 | In the past 3 months… How often have you felt safe and comfortable in and around your home? |
| OP4 | In the past 3 months… How often have you felt physically healthy? |
| OP5 | In the past 3 months… How often would you say you have been active or exercised – on a regular basis? |
|  | |
| **Factor: Wellbeing*,* control*,* network and meaningfulness** | |
| HO1 | In the past 3 months… How often have you felt mentally & emotionally healthy, happy and well? |
| OP6 | In the past 3 months… How often would you say you have felt purposefully occupied? |
| PE7 | In the past 3 months… How often have you felt that you have people/friends/loved ones who can support you if you need it? |
| PE8 | In the past 3 months… How regularly have you taken part in community/group activities? |
| PE9 | In the past 3 months… How often have you felt that you have been able to value and respect yourself? |
| EM10 | In the past 3 months… How often have you felt involved in the decisions that affect your life? |
| EM11 | In the past 3 months… How often have you felt in control and able to manage your life? |
| EM12 | In the past 3 months… How often have you felt hopeful for the future? |
| ^HO: Home, OP: opportunity, PE: people, EM: empowerment (Ion et al., 2013; Monger et al., 2013).^ | |

# REFERENCES

Huber, M., van Vliet, M., Giezenberg, M., Winkens, B., Heerkens, Y., Dagnelie, P. C., & Knottnerus, J. A. (2016). Towards a patient-centred operationalisation of the new dynamic concept of health. *British Medical Journal open*, *6*(1), 1–12. <https://doi.org/10.1136/bmjopen-2015010091> 10.1136/bmjopen-2015-010091

Ion, R., Monger, B., Hardie, S., Henderson, N., Cumming, J., Hardie, S., & Cumming, J. (2013). A tool to measure progess and Outcome in Recovery. *British Journal of Mental Health Nursing*, *2*, 211–215.

Monger, B., Hardie, S. M., Ion, R., Cumming, J., & Henderson, N. (2013). The Individual Recovery Outcomes Counter: preliminary validation of a personal recovery measure. *The Psychiatrist*, *37*(7), 221–227. <https://doi.org/10.1192/pb.bp.112.041889>

van Druten, V. P., Metz, M. J., Mathijssen, J. J. P., van de Mheen, D., van Vliet, M., Rudd, B., de Vries, E., & Nahar van - Venrooij, L. M. W. (2024). Measuring Positive Health using the My Positive Health (MPH) and Individual Recovery Outcomes Counter (I.ROC) Dialogue Tools: A Panel Study on Measurement Properties in a Representative General Dutch Population. *Applied research in quality of life*, *19*(5), 2825–2846. <https://doi.org/10.1007/s11482-024-10356-3>
